# Skin temperature variability is an independent predictor of survival in patients with cirrhosis

**DOI:** 10.14814/phy2.14452

**Published:** 2020-06-19

**Authors:** Matteo Bottaro, Noor‐Ul‐Hoda Abid, Ilias El‐Azizi, Joseph Hallett, Anita Koranteng, Chiara Formentin, Sara Montagnese, Ali R. Mani

**Affiliations:** ^1^ Department of Medicine University of Padova Padova Italy; ^2^ Network Physiology Lab Division of Medicine UCL London UK

**Keywords:** cirrhosis, heart rate variability, Poincaré plot, survival, temperature variability, thermoregulation

## Abstract

**Background:**

Cirrhosis is a disease with multisystem involvement. It has been documented that patients with cirrhosis exhibit abnormal patterns of fluctuation in their body temperature. However, the clinical significance of this phenomenon is not well understood. The aim of this study was to determine if temperature variability analysis can predict survival in patients with cirrhosis.

**Methods:**

Thirty eight inpatients with cirrhosis were enrolled in the study. Wireless temperature sensors were used to record patients’ proximal skin temperature for 24 hr. The pattern of proximal temperature fluctuation was assessed using the extended Poincaré plot to measure short‐term and long‐term proximal temperature variability (PTV). Patients were followed up for 12 months, and information was collected on the occurrence of death/liver transplantation.

**Results:**

During the follow‐up period, 15 patients (39%) died or underwent transplantation for hepatic decompensation. Basal proximal skin temperature absolute values were comparable in survivors and nonsurvivors. However, nonsurvivors showed a significant reduction in both short‐term and long‐term HRV indices. Cox regression analysis showed that both short‐term and long‐term PTV indices could predict survival in these patients. However, only measures of short‐term PTV were shown to be independent of the severity of hepatic failure in predicting survival. Finally, the prognostic value of short‐term PTV was also independent of heart rate variability, that is, a measure of autonomic dysfunction.

**Conclusion:**

Changes in the pattern of patients’ temperature fluctuations, rather than their absolute values, hold key prognostic information, suggesting that impaired thermoregulation may play an important role in the pathophysiology of cirrhosis.

## Graphical abstract

This report provides evidence that the pattern of skin temperature fluctuations predicts survival in patients with chronic liver disease independently of standard prognostic indicators (i.e. markers of the severity of liver failure and cardiac autonomic dysfunction). This finding extends our knowledge about the potential application of physio‐markers in the clinical assessment of patients with cirrhosis for organ transplant allocation.

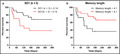

## 1 INTRODUCTION

Liver cirrhosis is a global health burden, whereby more than 1 million deaths occur worldwide annually (Asrani, Devarbhavi, Eaton, & Kamath, 2019; Mokdad et al., 2014). It is the ultimate outcome of chronic repetitive injury and inflammation to the liver, resulting in fibrosis and the formation of regenerative nodules, which replace the normal liver architecture. Although the pathology commences within the liver, cirrhosis results in the functional impairment of other organs and systems. Therefore, extrahepatic manifestations of cirrhosis such as fluid retention, hepatic encephalopathy, hyperdynamic circulation, hepatorenal syndrome, and immune dysfunction are common in patients with cirrhosis and are often the cause of hospital admission and mortality (Dong & Karvellas, 2019; Møller & Bendtsen, 2015).

Thermoregulation is a crucial homeostatic process, involved in maintaining our core body temperature within a narrow range (~2°C) (Tansey, Johnson, & Johnson, 2015). However, analysis of our skin temperature reveals that it possesses inherent variability, which exhibits short‐term (minute‐to‐minute) and long‐term (circadian) changes, whose functional significance may be to assist in the optimal control of our core body temperature in response to temperature changes in our external environment (Freeman & Linder, 1934; Papaioannou, Chouvarda, Maglaveras, & Pneumatikos, 2012; Satti et al., 2019). The short‐term variability in our body temperature is also reflective of the diverse range of autonomic, endocrine, and metabolic stimuli feeding into the hypothalamus and may also be reflective of the activity of pyrogenic cytokines released during systemic inflammation (Palanisamy, 2012; Satti et al., 2019; Tansey et al., 2015).

A variety of different computational methods have been developed to analyze fluctuations in patients’ body temperature, as well as experimental models (Ahmed et al., 2010; Mani, Mazloom, Haddadian, & Montagnese, 2018; Papaioannou et al., 2012; Satti et al., 2019). It appears that body temperature fluctuation analysis can provide crucial information regarding the integrity of one's thermoregulatory system (Mani et al., 2018; Satti et al., 2019). Recent studies have indicated that the pattern of skin temperature fluctuations has a prognostic value in critically ill patients (Cuesta et al., 2007; Papaioannou, Chouvarda, Maglaveras, Baltopoulos, & Pneumatikos, 2013; Papaioannou et al., 2012; Papaioannou, Sertaridou, Chouvarda, Kolios, & Pneumatikos, 2019; Varela et al., 2006). Our body temperature exhibits an inherent fractal‐like pattern in its fluctuations and hence exhibits a certain degree of “complexity,” such that alterations in the extent of this complexity are associated with poor prognosis in patients with multiple‐organ failure (Varela et al., 2006). Furthermore, Papaioannou et al. revealed that metrics associated with measuring entropy (an index of irregularity and disorder) of patients’ skin temperature variability in a critically ill cohort of septic patients exhibited better prognostic accuracy than the SOFA (sequential organ failure assessment) score currently used in the clinical setting (Papaioannou et al., 2013).

Recent research has revealed that cirrhotic patients exhibit a different pattern in their skin temperature fluctuations compared to healthy individuals (Garrido et al., 2017; Satti et al., 2019). In addition, Garrido et al. have proposed evidence to suggest that cirrhotic patients manifest an abnormal circadian rhythm of temperature, and also exhibit substantially altered proximal and distal skin temperature profiles (Garrido et al., 2017). This has been postulated to be due to systemic vasodilation, as a result of the extensive release of endogenous vasodilators or due to the autonomic dysfunction observed in patients with cirrhosis, thus highlighting that cirrhotic patients’ temperature variability profile does indeed change and is suggestive of altered thermoregulatory function, compared to healthy individuals. Mani et al. also revealed that cirrhotic rats exhibit greater entropy in their core body temperature, compared to control rats, possibly indicative of an increased amount of coupling and information processing in the thermoregulatory system (Mani et al., 2018). Based on these recent studies there is evidence to suggest that cirrhosis is associated with an altered pattern in the fluctuations of patients’ body temperature profile, which may indeed be correlated with multisystem involvement of cirrhosis. The mechanism of this phenomenon is currently not well understood. Moreover, it is not known whether temperature variability analysis has a prognostic value in the context of cirrhosis. Recent studies have shown that physiological markers such as heart rate variability (HRV) predict mortality in patients with cirrhosis, independently of other indices of liver failure such as the Child‐Pugh and MELD (Model for End‐Stage Liver Disease) scores (Bhogal et al., 2019; Satti et al., 2019). It appears that systemic inflammation is involved in the pathogenesis of poor prognosis in cirrhosis (Clària et al., 2016). Systemic inflammation is linked with reduced HRV (Bhogal et al., 2019; Haddadian et al., 2013) and altered body temperature dynamics in chronic liver failure (Mani et al., 2018), so it is plausible to assess these physio‐markers in prediction of poor prognosis in cirrhosis.

The present study was designed to determine whether measures of skin temperature variability can predict mortality in patients with cirrhosis and whether this is independent of the severity of liver failure and HRV.

## 2 METHODS

### 2.1 Ethics statement

This study was approved by the ethics committee of the University Hospital of Padova, (code: 4196/AO/17). Written informed consent was provided by all patients involved in the study. Data were recorded according to the Data Protection Act.

### 2.2 Patients population and measurement of proximal skin temperature

38 patients with cirrhosis were admitted to Clinica Medica 5 at the Padova University Hospital and were included in the study based on the inclusion and exclusion criteria. Their etiology was established through conventional clinical and pathological findings, and Child‐Pugh and MELD scores were obtained. Inclusion criteria: hospital admission and signed informed consent. Exclusion criteria: Fever and antibiotic treatment in the preceding 3 days.

The mean age (±1 *SD*) in the study was 64.62 years ± 10.4 years. The study took place between 06/04/2017 and 02/02/2019 (date of the first temperature recording–last survey date after follow‐up for survival analysis).

**FIGURE 1:**
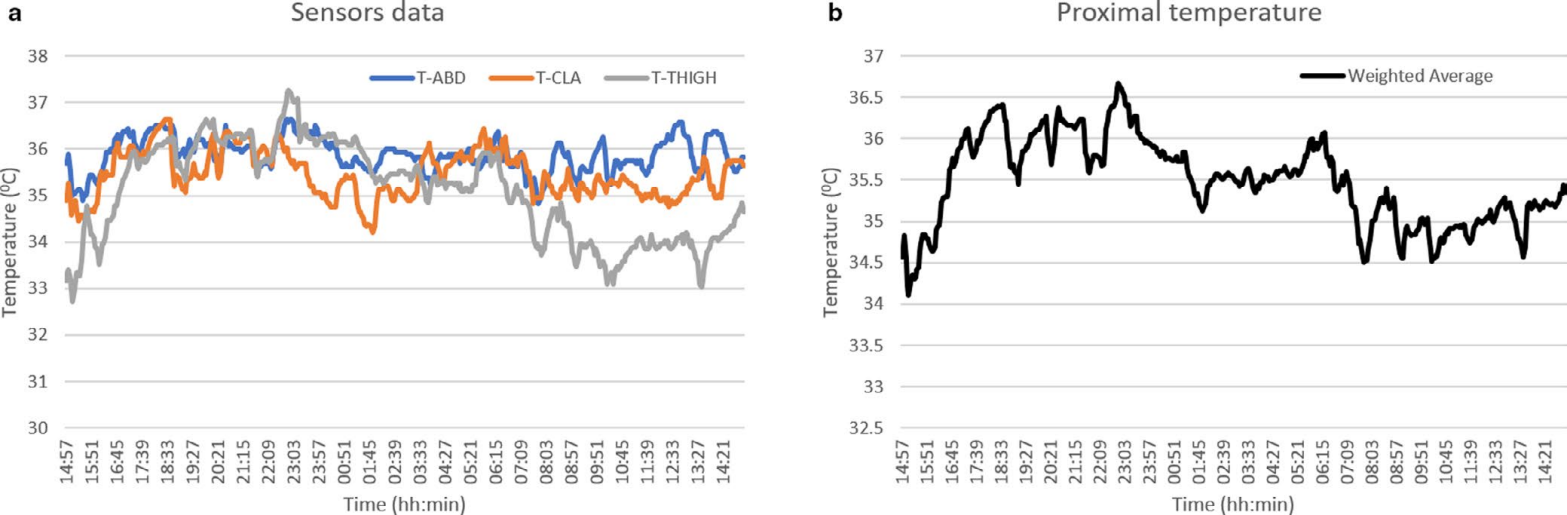
A sample of a 24‐hr temperature recording using three proximal sensors (a) and their weighted average (b)

Three wireless temperature probes (iButtons, model no. DS1922L‐F5, Maxim Integrated) were placed on the abdomen, intra‐clavicular area, and mid‐thigh of patients for 24 hr, as described by Longato et al. (Longato et al., 2017). The sampling rate of the data logger was 1 signal per 3 min. The following formula was used to calculate proximal temperature time series based on weighted average of the three proximal sensors placed on the patients as described earlier (Longato et al., 2017) (an example of which is represented in Figure 1):

$$T_{\text{Prox}}=0.379\;T_{\text{Abdomen}}+0.262\;T_{\text{Intra}\;-\;\text{clavicular}}+0.359\;T_{\text{Mid}\;-\;\text{thigh}}$$

Electrocardiograms (ECG) were recorded using a wireless probe (Actiwave Cardio, CamNtech) for 24 hr. The ECG sampling rate was 256 Hz. These ECG files were then subsequently used to calculate the HRV indices which are known physio‐markers of predicting survival in patients with cirrhosis.

### 2.3 Patient follow‐up

**FIGURE 2:**
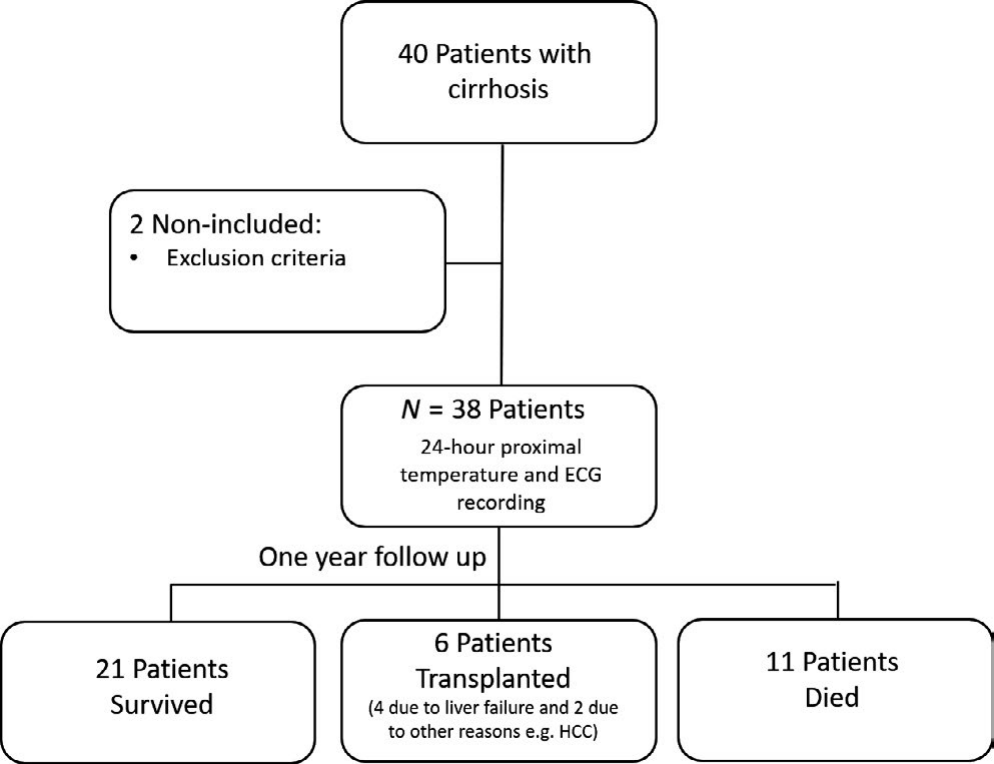
A flowchart of the procedures in the study

Patients were followed up for 12 months and information regarding the occurrence of death or liver transplantation was collected during this follow‐up period. Patients who underwent transplantation for hepatic failure were considered “nonsurvivors” on the day of transplantation (as they were in immediate need of a new liver and would not survive without it) while patients who underwent transplantation for hepatocellular carcinoma were censored on the date of transplantation. During the follow‐up period if contact was lost from a patient before the pre‐defined time (12 months) was up, the patient was also censored on the date of the latest available information. A flowchart of the procedures involved in the study is outlined in Figure 2.

### 2.4 Temperature variability (TV) analysis

The standard deviation of the proximal temperature (T_Prox_) time series obtained from patients was calculated as an index of the total PTV. In addition, nonlinear analytical methods such as the extended Poincaré plot, sample entropy, detrended fluctuation analysis, and memory length were used to quantify skin temperature dynamics and ultimately the PTV indices. All algorithms were computed using the MATLAB programming language (MathWorks). The following is a brief description of the analytical methods mentioned above used to assess patients’ PTV. Further details of these methods are delved in further in other literature (Peng, Havlin, Stanley, & Goldberger, 1995; Richman & Moorman, 2000; Satti et al., 2019; Shirazi et al., 2013).

#### 2.4.1 The Poincaré plot

The *Poincaré plot* is a method used to visualize and quantify the correlation between two consecutive data points in a time series (Mani et al., 2009). As the dynamics of the fluctuations exhibited in skin temperature profiles possess long‐term correlation and thus memory, the extended Poincaré plot considers the correlation between sequential data points in a time series (i.e., T_n_ vs. T_n + k_, where k [also known as the ‘lag’] can be any discrete point along the time series), rather than between two consecutive points (see Appendix 1 for a graphical representation). Thus, it provides more information about the dynamics of skin temperature fluctuations and can be used to estimate the short‐term and long‐term PTV possessed in the patients’ time series (Satti et al., 2019). Based on our prior knowledge, it appears that short‐term PTV may reflect skin temperature variation probably due to myogenic and neurogenic inputs to peripheral microvasculature. Long‐term‐PTV may reflect fluctuation with a longer timescale within the 24‐hr circadian variation.

#### 2.4.2 Sample entropy

*Sample entropy* is a tool that quantifies the degree of “disorder” and therefore the extent of irregularity present in a time series. This “irregularity” is the inverse of the amount of regularity present in the time series, and is quantified by calculating the negative logarithmic likelihood of the probability that points in each epoch (period of time) of window length “m” follow a similar structure and pattern, with tolerance “r” later on in the time series. In this study “m” was set at 2 and “r” at 0.2 as described by Richman and Moorman (Richman & Moorman, 2000). Sample entropy has been shown to reflect the complexity of a physiological time series (Richman & Moorman, 2000) and its reduction often indicates isolation of the regulatory components of a physiological system (Pincus, 1994).

#### 2.4.3 *Detrended fluctuation analysis* (DFA)

*Detrended fluctuation analysis* (DFA) was carried out to study the fractal‐like behavior of 24‐hr temperature time series. In this analysis the data are split into boxes of various lengths (n) and this is plotted against the *F*(n), which is the in different scales (n). The slope of the resulting log–log graph is known as “scaling exponent” of α which indicates the type of fractal‐like dynamics present in the physiological signal. DFA analysis has been used in physiological time series to show deviation from a random time series. The scaling exponent (α) for a random time series is expected to be 0.5 (Peng et al., 1995). Thus, any divergence from this value toward 1 indicates deviation from random fluctuations.

#### 2.4.4 Memory length

The concept of *memory length* possessed within a physiological time series was introduced by Shirazi et al. (Shirazi et al., 2013). Memory in a physiological time series is defined as a time period, during which “rare” events within a physiological time series do not appear randomly and may be repeated for a certain length of time (Shirazi et al., 2013). This method is based on the calculation of the distribution of the time elapsed to observe a rare event (e.g., a jump or drop) in a time series (Ebadi, Shirazi, Mani, & Jafari, 2011;2011.). A jump in a temperature time series is a point along the time series that shows + 3σ degree rise in skin temperature, whereby σ is the standard deviation of temperature time series. Alternatively, a drop in the time series is a rare event defined as −3σ degree changes in skin temperature and can be used to calculate the memory length (−3σ) (Shirazi et al., 2013; Taghipour et al., 2016). It is harder to control a system with prolonged dependency on its past. Thus, prolongation of memory is indirectly linked to a reduced controllability in a complex system (Mazloom, Shirazi, Hajizadeh, Dehpour, & Mani, 2014); therefore, memory length was computed for estimation of controllability of skin temperature variations in the present study.

### 2.5 Heart rate variability (HRV) analysis

The R peaks were detected using ECGs on patients and their subsequent inter‐beat interval time series was then generated by using HRV analysis software (version 1.1.) (Pichot, Roche, Celle, Barthélémy, & Chouchou, 2016). Artifacts were removed from these 24‐hr R–R interval time series using a filter embedded in the HRV analysis software. The following HRV indices were then computed using algorithms written in MATLAB as described previously (Bhogal et al., 2019):

#### 2.5.1 SDNN

Standard deviation of the R–R intervals, as a measure of the total HRV.

#### 2.5.2 cSDNN

It is known that SDNN is inversely related to mean heart rate in healthy individuals (Monfredi et al., 2014). Therefore, some investigators normalize SDNN for heart rate using nonlinear models (Monfredi et al., 2014). The corrected SDNN (cSDNN) for patients’ heart rate was calculated using the following formula (Monfredi et al., 2014): $\text{cSDNN}=\frac{\text{SDNN}}{e^{-}\frac{\text{Heart rate}}{58.8}}$.

#### 2.5.3 SD1

Standard deviation perpendicular to the line of identity on the extended Poincaré plot quantifies the short‐term variability exhibited in patients’ cardiac rhythm.

#### 2.5.4 SD2

Standard deviation parallel to the line of identity on the extended Poincaré plot quantifies the long‐term variability exhibited in patients’ cardiac rhythm.

#### 2.5.5 Spectral indices

Spectral analysis of the R–R interval time series was carried out by fast Fourier transformation. Three bands were identified: VLF: a very low‐frequency component (0–0.04 Hz), LF: a low‐frequency component (0.04–0.15 Hz), and HF: a high‐frequency component (0.15–0.4 Hz).

#### 2.5.6 Sample entropy

The degree of regularity and subsequently the degree of irregularity of the inter‐beat interval time series were calculated as described above using the parameters m = 2 and r = 0.2, as recommended by Richman and Moorman (Richman & Moorman, 2000).

#### 2.5.7 Fractal‐like exponents

Cardiac cycles exhibit fractal‐like dynamics which may be affected by systemic inflammation in cirrhosis (Haddadian et al., 2013). DFA was employed to calculate the scaling exponent (α) as described above. Since 24‐hr R–R interval time series exhibit a cross‐over phenomenon, α was separately calculated for short windows (epochs of times with scale ≤ 16) and long windows (scale > 16). Thus, we calculated two different values of α, α1, and α2, which reflect short‐term and long‐term fractal‐like exponents, respectively (Peng et al., 1995).

### 2.6 Statistical analysis

TV or HRV indices of survivors and nonsurvivors were compared using either Student's *t* test or the nonparametric Mann–Whitney U‐test according to the distribution of the variables involved. Data with two dependent variables (i.e., group [survivors vs. nonsurvivors] and lag (k)) were analyzed using the two‐way ANOVA. A value of *p* < .05 was considered statistically significant.

#### 2.6.1 Survival analysis

Cox proportional hazards regression was used for survival analysis. In this analysis, the Cox regression coefficient (β) and the hazard ratio (e^β^) were calculated and the p‐value for testing the null hypothesis (β = 0, hazard ratio = 1) was determined using the Wald test. The bivariate Cox regression model was used to test if the prediction of mortality by PTV indices is independent of the severity of hepatic failure. The ROC curve was used to choose the best cut‐off points with the highest sensitivity and specificity at predicting survival for the categorization of patients in the Kaplan–Meier graph based on their PTV indices. The log‐rank (Mantel–Cox) test was used for survival analysis in the Kaplan–Meier graph. Statistical analysis was conducted using the SPSS package (IBM). Values of *p* < .05 were considered statistically significant.

## 3 RESULTS

### 3.1 Participants

Following 24‐hr skin temperature and ECG recordings, 38 patients were followed up for 12 months. During the follow‐up period: 21 patients survived, 11 patients (29%) died, and six patients underwent transplantation of whom four underwent transplantation due to liver failure and 2 for the treatment of hepatocellular carcinoma (Figure 2). The most common causes of hospitalization were hepatic encephalopathy (15/38), tense ascites (10/38), and hepatorenal syndrome (5/38).

The general characteristics of the study population are presented in Table 1. There was no significant difference between the age and gender of survivors and nonsurvivors; however, both the MELD and Child‐Pugh scores were significantly different (*p* < .05).

**TABLE 1:** Mean characteristics of the study population

	Survivors	Nonsurvivors	*p*‐value
Number	23	15	—
Gender (male/female)	19/4	11/4	.637
Age	64.0 ± 2.3	65.0 ± 2.5	.768
MELD	17.82 ± 1.76	23.76 ± 1.98	**.031**
Child‐Pugh	8.90 ± 0.43	10.54 ± 0.58	**.022**

Note

Data are expressed as mean ± *SEM* with the exception of gender which is expressed as the ratio of male/female. The level of significance was set at *p* < .05. Fisher's exact test was used to compare the genders.

Bold values represent when *p* < .05.

### 3.2 Temperature variability data

**FIGURE 3:**
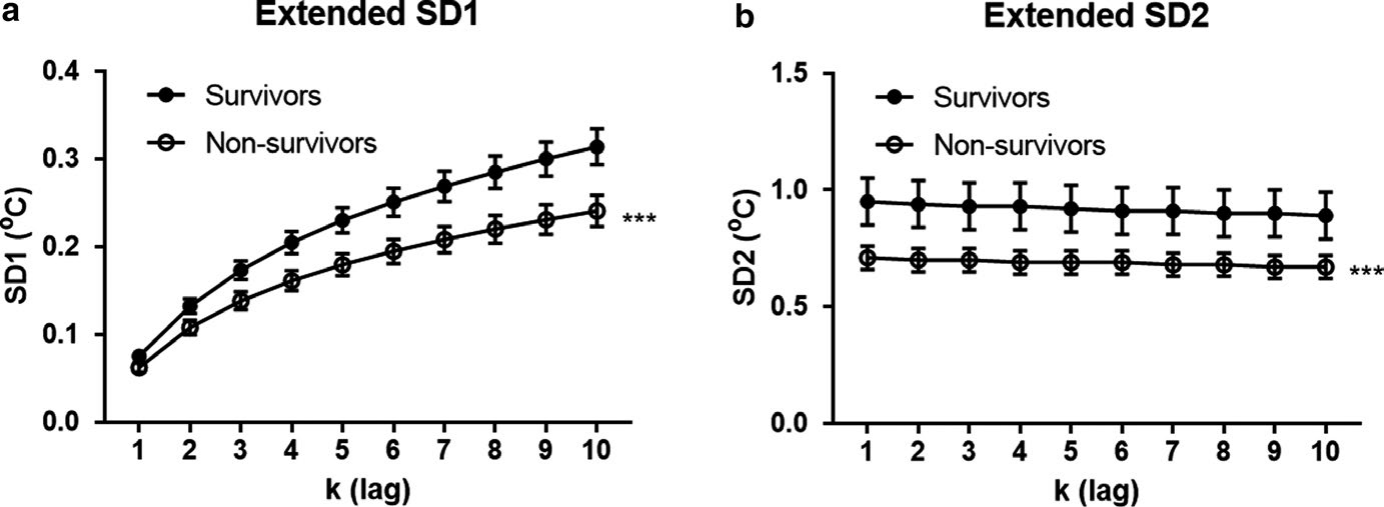
SD1 (a) and SD2 (b) derived from the extended Poincaré plot depicting the relationship between proximal temperature fluctuations (T_n_ versus T_n + k_, whereby k = {1, 2, …, 10}) in those who survived with cirrhosis in comparison to those who did not (nonsurvivors). ***p < .0001 based on the analysis of the effect of group (survivors vs. nonsurvivors) in a two‐way ANOVA

Table 2 compares mean proximal temperature and PTV indices between survivors and nonsurvivors. The mean proximal temperature and standard deviation of the 24‐hr temperature time series were similar in the surviving and nonsurviving patient groups. There was a significant reduction in the short‐term (SD1 [k = 2 and 3]) and long‐term PTV (SD2 [k = 1, 2 and 3]) in nonsurvivors compared to those who survived when the extended Poincaré plot was used for the quantification of patients’ PTV. As shown in Figure 3a, the analysis of the data's short‐term variability (SD1) enabled us to distinguish between survivors and nonsurvivors (*F*_group_ = 54.97, *p* < .0001) at steps (k) (*F*_lag_ = 41.61, *p* < .0001); however, there was no interaction between the two (group and steps, *F_interaction_* = 0.902, *p* = .523). As shown in Figure 3b, the analysis of the data's long‐term variability (SD2) also separated survivors from nonsurvivors. In fact, there was a significant difference between patient groups (*F*_group_ = 34.23, *p* < .0001) but not between steps (k), meaning that the declination trend is not significant (*F_l_*_ag_ = 0.066, *p* = .999).

**TABLE 2:** Mean proximal temperature characteristics and temperature variability indices of the study population

	Survivors	Nonsurvivors	*p*‐value
Number	23	15	—
Mean proximal temperature (^°^C)	35.05 ± 0.16	35.36 ± 0.19	.220
*SD* of proximal temperature (^°^C)	0.674 ± 0.071	0.504 ± 0.045	.065
SD1 (k = 1) (^°^C)	0.075 ± 0.005	0.062 ± 0.005	.068
SD1 (k = 2) (^°^C)	0.132 ± 0.008	0.108 ± 0.008	**.049**
SD1 (k = 3) (^°^C)	0.173 ± 0.011	0.138 ± 0.010	**.026**
SD2 (k = 1) (^°^C))	0.949 ± 0.101	0.710 ± 0.053	**.043**
SD2 (k = 2) (^°^C)	0.941 ± 0.102	0.708 ± 0.050	**.045**
SD2 (k = 3) (^°^C)	0.933 ± 0.102	0.699 ± 0.050	**.047**
Sample Entropy	0.461 ± 0.031	0.448 ± 0.029	.774
Scaling exponent (α)	1.40 ± 0.02	1.37 ± 0.03	.383
Memory length (+3σ)	4.30 ± 0.14	6.12 ± 1.45	.230
Memory length (−3σ)	4.17 ± 0.08	6.73 ± 0.87	**.010**

Note

Data are expressed as mean ± *SEM*. The level of significance was set at *p* < .05.

Bold values represent when *p* < .05.

The sample entropy and scaling exponents (α) of the 24‐hr temperature time series were comparable in survivors and nonsurvivors (Table 2). However, the memory length for observing a drop in proximal skin temperature (memory length [−3σ]) was significantly prolonged in nonsurvivors in comparison with survivors (*p* < .01). There was however no significant difference in the memory length for observing a rise in proximal skin temperature (memory length [+3σ]) as shown in Table 2.

### 3.3 PTV predictors of mortality

The first part of the survival analysis was to determine which PTV parameters from Table 2 were linked with mortality according to Cox regression analysis. From the variables listed in Table 3, it is evident that only four PTV indices could predict mortality. As expected, both the MELD and Child‐Pugh scores could predict mortality. Interestingly, indices of short‐term PTV in the extended Poincaré plot (SD1 [k = 1, 2 and 3]) came up as significant. These indices had hazard ratios below 1 which indicate that an increase in short‐term temperature variability is associated with a reduction in mortality and, conversely, a decrease in short‐term temperature variability is associated with an increase in mortality. In addition, the memory length (−3σ) is shown to be a predictor of mortality (hazard ratio: 1.259, *p* < .001, Table 3) which indicates that an increase in memory length is associated with higher patient mortality.

**TABLE 3:** Predictive effect of age, hepatic dysfunction, and temperature variability indices on one‐year mortality

	β	*SEM*	Hazard ratio	*p*‐value
Age	0.007	0.024	1.007	.778
MELD	0.069	0.027	1.072	**.009**
Child‐Pugh	0.303	0.122	1.354	**.013**
Mean proximal temperature	0.325	0.314	1.384	.301
*SD* of proximal temperature	−2.570	1.504	0.077	.088
SD1 (k = 1)	−36.914	18.129	0.000	**.041**
SD1 (k = 2)	−21.591	10.020	0.000	**.031**
SD1 (k = 3)	−17.996	7.630	0.000	**.018**
SD2 (k = 1)	−1.808	1.064	0.164	.089
SD2 (k = 2)	−1.777	1.060	0.169	.094
SD2 (k = 3)	−1.744	1.056	0.175	.099
Sample Entropy	−0.885	1.784	0.413	.620
Scaling exponent (α)	−1.389	2.148	0.250	.519
Memory length (+3σ)	0.066	0.095	1.062	.181
Memory length (−3σ)	0.231	0.068	1.259	**.001**

Note

β is the coefficient of Cox regression analysis. *SEM* is the standard error of the mean of β, Hazard ratio = Exp (β) = *e* ^β^. The level of significance was set at p < .05 (bold values).

### 3.4 Analysis of the independence of PTV parameters from markers of liver failure in predicting mortality

Using the four PTV indices that were significant in Cox regression analysis, we combined these indices with a measure of disease severity (MELD or Child‐Pugh) to determine if the ability of these PTV indices to predict mortality was independent of the severity of liver failure. Bivariate Cox regression analysis showed that SD1 (k = 2), SD1 (k = 3), and memory length (−3σ) were independent of MELD in predicting mortality in patients with cirrhosis (Table 4). This analysis was also repeated with the Child‐Pugh score and results were similar (Table 5).

**TABLE 4:** Independence of temperature variability indices from MELD score in predicting mortality in Cox bivariate regression analysis

	β	*SEM*	Hazard ratio	*p*‐value
SD1 (k = 1)	−31.767	16.640	0.000	.056
SD1 (k = 2)	−18.841	9.158	0.000	**.040**
SD1 (k = 3)	−16.028	6.966	0.000	**.021**
Memory length (−3σ)	0.195	0.071	1.216	**.006**

Note

MELD score was significant in the analysis when compared with the other variables listed in Cox bivariate regression analysis. The level of significance was set at p < .05 (bold values)

**TABLE 5:** Independence of temperature variability indices from Child‐Pugh score in predicting mortality in Cox bivariate regression analysis

	β	*SEM*	Hazard ratio	*p*‐value
SD1 (k = 1)	−33.028	16.944	0.000	.051
SD1 (k = 2)	−19.505	9.331	0.000	**.037**
SD1 (k = 3)	−16.682	7.123	0.000	**.019**
Memory length (−3σ)	0.204	0.068	1.226	**.003**

Note

Child‐Pugh score was significant in the analysis when compared with the other variables listed in Cox bivariate regression analysis. The level of significance was set at p < .05 (bold values)

### 3.5 Kaplan–Meier graphs based on PTV analysis

**FIGURE 4:**
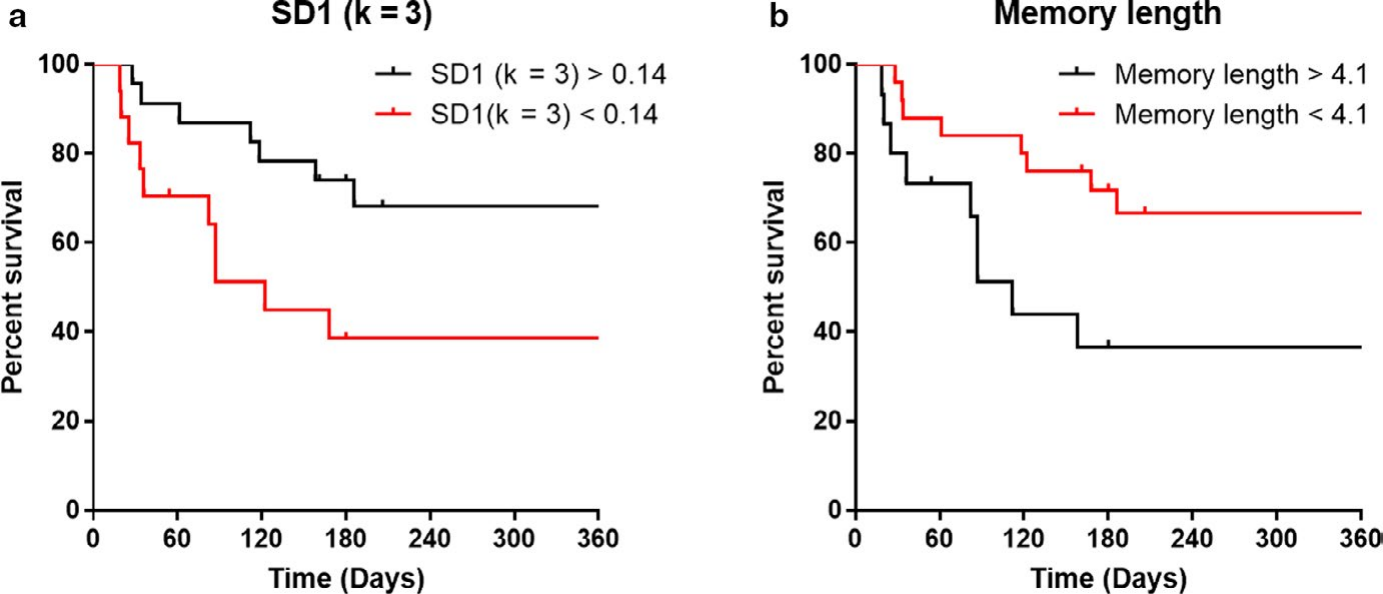
Kaplan–Meier graphs illustrating how temperature variability indices can predict survival in patients with cirrhosis. Survival graphs depicting the overall survival of patients with cirrhosis above and below the cut‐off value for SD1 (k = 3) or memory length. (a) SD1 (k = 3) (Log‐rank test, Chi square = 4.504, *p* < .05). (b) Memory length (−3σ) (Log‐rank test, Chi square = 4.481, *p* < .05)

Kaplan–Meier graphs were plotted for the two PTV indices that showed clear independence from MELD and Child‐Pugh: SD1 (k = 3) and memory length (−3σ). ROC curve analysis was then conducted to determine the cut‐off value that provided the best possible trade‐off of sensitivity and specificity (data are presented in Appendix 2). The cut‐off values for SD1 (k = 3) and memory length (−3σ) were 0.14°C and 4.1, respectively. These cut‐off values with the best sensitivity and specificity of these two parameters at predicting survival of patients with cirrhosis are depicted in the Kaplan–Meier graphs for the two variables, SD1 (k = 3) and memory length (−3σ) (Figure 4a,b respectively). The cut‐off value for patients’ SD1 (k = 3) clearly discriminates between those with poor prognosis whereby SD1 (k = 3) <0.14 compared to those with much better prognosis, whereby SD1 (k = 3) >0.14 and shows a marked difference in their overall one‐year survival 12 months post‐temperature recording (Log‐rank test, Chi square = 4.504, *p* = .0338) (Figure 4a). Similarly, the cut‐off value for patients’ memory length (−3σ) clearly discriminates between those with poor prognosis whereby their memory length (−3σ) >4.1, compared to those with much better prognosis, whereby their memory length (−3σ) < 4.1 and shows a marked difference in their overall one‐year survival 12 months post‐temperature recording (Log‐rank test, Chi square = 4.481, *p* = .0343).

### 3.6 HRV and survival in patients with liver cirrhosis

24‐hr ECG recordings were obtained and 24‐hr HRV indices calculated. The basal 24‐hr mean heart rate was comparable in survivors and nonsurvivors (76.6 ± 3.5 versus 72.9 ± 3.0, *p* = .459). Studying the HRV characteristics revealed that several parameters were significantly different between the two groups (Appendix 3). Total HRV indices (SDNN, cSDNN) were significantly lower in nonsurvivors (*p* < .05). As shown in Appendix 3, all indices of long‐term HRV (SD2 and VLF) exhibited a significant reduction in nonsurvivors. The long‐term scaling exponent (α2) was also significantly lower in nonsurvivors (*p* = .004).

From the variables listed in Appendix 3, it became evident that most of them could predict mortality in Cox regression analysis (Table 6). We then used bivariate Cox regression analysis to determine if the ability of these HRV indices to predict mortality was independent of the severity of liver failure, as indicated by patients’ MELD and Child‐Pugh score. Bivariate Cox regression analysis revealed that among the 24‐hr HRV indices, only the long‐term scaling exponent (α2) was independent of MELD (Table 7). This analysis was also repeated with the Child‐Pugh score and revealed that among the HRV indices tested; the long‐term HRV indices: SD2, VLF, and long‐term scaling exponent (α2) were all significant predictors of mortality, independent of Child‐Pugh score (data not shown).

**TABLE 6:** Predictive effect of 24‐hr heart rate variability (HRV) indices on one‐year mortality in patients with cirrhosis

	β	*SEM*	Hazard ratio	*p*‐value
Mean heart rate (bpm)	−0.016	0.019	0.984	.410
SDNN	−0.021	0.010	0.979	**.028**
cSDNN	−0.007	0.003	0.993	**.015**
SD1	0.028	0.015	1.029	.056
SD2	−0.017	0.007	0.983	**.016**
VLF	−0.000	0.000	0.999	**.013**
LF	0.001	0.000	1.001	.056
HF	0.001	0.001	1.001	**.007**
Sample entropy	0.983	0.541	2.671	.069
Short‐term scaling exponent (α_1_)	−1.632	0.885	0.195	.065
Long‐term scaling exponent (α_2_)	−4.509	1.559	0.011	**.004**

Note

β is the coefficient of Cox regression analysis. *SEM* is the standard error of the mean of β, Hazard ratio = Exp (β) = *e* ^β^. The level of significance was set at p < .05 (bold values)

**TABLE 7:** Independence of heart rate variability (HRV) indices from MELD score in predicting mortality in Cox bivariate regression analysis

	β	*SEM*	Hazard ratio	*p*‐value
SDNN	−0.014	0.010	0.986	.172
cSDNN	−0.005	0.003	0.995	.120
SD2	−0.012	0.007	0.988	.107
VLF	−0.000	0.000	0.999	.062
Long‐term scaling exponent (α_2_)	−6.630	2.064	0.001	**.001**

Note

MELD score was significant in the analysis when compared with the other variables listed in Cox bivariate regression analysis. The level of significance was set at p < .05 (bold value).

**FIGURE 5:**
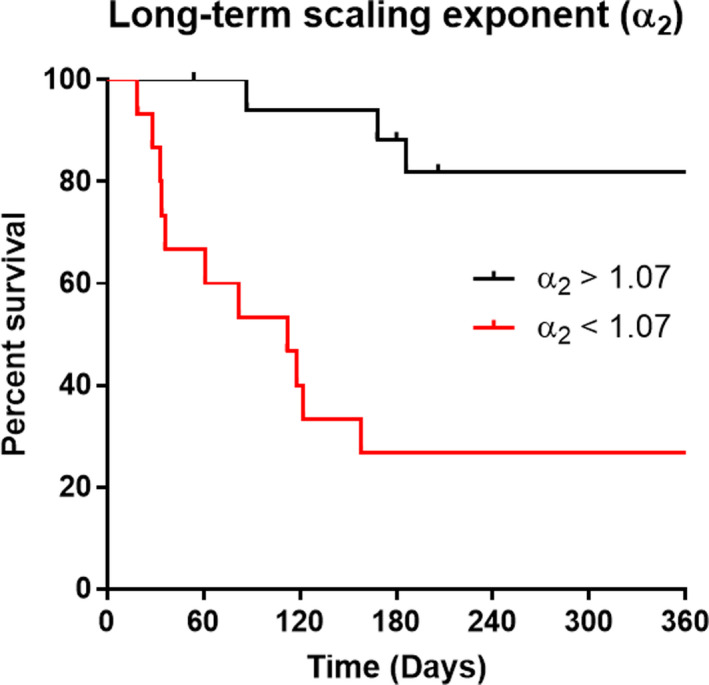
Kaplan–Meier graphs illustrating how the long‐term scaling exponent (α2), a HRV index can predict survival in patients with cirrhosis. Survival graph depicting the overall survival for patients with cirrhosis above and below α2 cut off values (Log‐rank test, Chi square = 13.08, *p* = .0003)

As the long‐term scaling exponent (α2) was the only 24‐hr HRV index that showed independence from both the MELD and Child‐Pugh score, a Kaplan–Meier graph was plotted for this index. As described earlier, ROC curve analysis was conducted to determine the cut‐off value for α2 (1.07). Figure 5 depicts that the cut‐off value for patients’ long‐term scaling exponent (α2) clearly discriminates between those with poor prognosis whereby α2 < 1.07 compared to those with much better prognosis, whereby α2 > 1.07 and shows a marked difference in their overall one‐year survival 12 months post‐temperature recording (Log‐rank test, Chi square = 13.08, *p* = .0003).

### 3.7 Analysis of the independence of PTV indices from HRV indices in predicting mortality

Bivariate Cox regression analysis was then conducted to determine whether the two PTV indices (SD1 [k = 3] and memory length [−3σ]) as well as the HRV index, α2 (that were all independent of MELD and the Child‐Pugh score) could themselves predict mortality independently of each other. Bivariate Cox regression analysis showed that SD1 (k = 3) (a PTV index) and α2 (an HRV index) could predict mortality independently of each other (Table 8A). The same result was true for the PTV index, memory length (−3σ) and the HRV index, α2 (Table 8B). This suggests that the PTV and HRV indices can independently predict one‐year mortality in patients with cirrhosis, 12 months post‐temperature recording.

**TABLE 8:** Independence of temperature variability (TV) from heart rate variability (HRV) indices in predicting mortality in Cox bivariate regression analysis.

	β	*SEM*	Hazard ratio	*p*‐value
A				
TV index				
SD1 (k = 3)	−18.921	8.807	0.000	**.032**
HRV index				
Long‐term scaling exponent (α_2_)	−4.631	1.657	0.010	**.005**
B					
TV index					
Memory length (−3σ)	0.172	0.071	1.188	**.015**	
HRV index					
Long‐term scaling exponent (α_2_)	−4.057	1.667	0.017	**.015**	

Bold values represent when *p* < .05.

## 4 DISCUSSION

This study was aimed at determining the prognostic value of skin temperature variability in patients with cirrhosis. We discovered that some PTV indices are predictors of survival in patients with cirrhosis. Specifically, the PTV indices quantifying the memory length (−3σ) and short‐term variability (SD1 [k = 3]) exhibited in patients’ time series predicted mortality over the 12 months post‐temperature recording, independently of the degree of liver dysfunction. Furthermore, the long‐term scaling exponent (α2) HRV index could also predict survival in patients with cirrhosis independently of their MELD score. We then compared the prognostic value of these HRV and PTV indices in this patient population and found that although both physio‐markers predict mortality, their prognostic values were independent. To the best of our knowledge these results are the first report, which make a link between the pattern of skin temperature fluctuations and the prognosis of patients within the context of chronic liver disease. These results also extend our knowledge about the potential application of physio‐markers in the clinical assessment of patients with cirrhosis (Mani et al., 2009; Montagnese, De Rui, et al., 2015; Montagnese, Jackson, & Morgan, 2007; Olesen et al., 2016). There has been an interest in recent years in the application of HRV for monitoring patients with cirrhosis awaiting liver transplantation (Bhogal et al., 2019; Bhogal, Montagnese, & Mani, 2018; Chan, Yeh, & Sun, 2017; Jansen et al., 2019; Xiong, Faes, & Ch, 2017). However, this study reveals that HRV is not the only physio‐marker that provides useful information about patient outcomes and our results suggest that the noninvasive assessment of patients’ thermoregulation using PTV analysis may add value in assessing the broader manifestations exhibited in patients with cirrhosis.

Absolute values of temperature are not informative of the underlying processing of patients’ autonomic thermoregulatory circuits. Nevertheless, PTV analysis aims to elucidate this by quantifying changes in the inherent dynamics of our thermoregulatory system. Historically, body temperature has only been given significance in the context of fever; however, the integrity and performance of the thermoregulatory system can be affected in a multitude of diseases (Mani et al., 2018; Papaioannou et al., 2012). Systemic disorders can affect the dynamics of heat loss and cellular heat production via multiple mechanisms such as vasodilatation (Charkoudian, 2010) and mitochondrial dysfunction (Argyropoulos & Harper, 2002), respectively. Furthermore, many systemic diseases such as liver cirrhosis have a significant impact on one's circadian rhythm (Blei & Zee, 1998; De Rui et al., 2015; Montagnese, Middleton, et al., 2015; Montagnese, Middleton, Mani, Skene, & Morgan, 2010) and 24‐hr body temperature fluctuations (Garrido et al., 2017). Alterations in skin temperature dynamics and its link with poor outcome is documented in acute clinical settings such as sepsis (Papaioannou et al., 2012, 2013, 2019). The novelty of our report is that in this study, PTV analysis was shown to predict survival in a chronic illness (cirrhosis), independently of standard prognostic indicators (MELD/Child‐Pugh). None of the patients in the present study had fever. Additionally, the basal proximal skin temperature of both survivors and nonsurvivors was identical, further emphasizing the utility and value of analyzing the variability exhibited in patients’ temperature profiles, rather than measuring absolute values. Using the extended Poincaré analysis, Satti et al. had previously reported that hospitalized inpatients with cirrhosis exhibit lower short‐term PTV compared with healthy volunteers and outpatients with cirrhosis (Satti et al., 2019). Our results expand on this finding and reveal that lower short‐term PTV is indeed linked with poor prognosis. The exact mechanistic basis by which cirrhosis affects short‐term PTV is unknown. Short‐term PTV reflects skin temperature variation probably due to myogenic and neurogenic inputs to peripheral microvasculature. Cirrhosis is associated with hyperdynamic circulation and peripheral vasodilatation which potentially affect heat loss as well as metabolic rate (Bolognesi, Di Pascoli, Verardo, & Gatta, 2014; Müller, Böker, & Selberg, 1994; Müller et al., 1999). The extent of vasodilatation and its dynamics is not routinely measured while monitoring patients with cirrhosis. Nonetheless, it is reasonable to postulate that reduced short‐term PTV may be the result of impaired autonomic control of peripheral vasculature in patients with cirrhosis. Additionally, the short‐term PTV in those that survived was greater than in those who exhibited a poor prognosis. This suggests that an increase in short‐term PTV may be reflective of increased coupling of autonomic thermoregulatory circuits and hence a greater engagement of the control system in trying to combat the extensive heat loss due to the systemic vasodilation. Thus, implying that a decrease in patients’ short‐term PTV associated with poor prognosis may indeed be reflective of decreased coupling, and engagement of their autonomic thermoregulatory system. However, whether there's a causal link requires further research to validate. There are also other reasonable mechanisms attempting to explain why reduced short‐term PTV is associated with poorer prognosis such as the hypothesis of impaired cellular heat production dynamics in nonsurvivors, due to impaired mitochondrial function (Mansouri, Gattolliat, & Asselah, 2018; Moreau et al., 2020) or hormone abnormalities exhibited in chronic liver disease (Patira, Salgiya, & Agrawal, 2019; Vincken, Reynaert, Schiettecatte, Kaufman, & Velkeniers, 2017). Systemic inflammation in patients with cirrhosis is energetically expensive and its metabolic costs may result in reduced heat production as well as multi‐organ failure/poor prognosis in patients with liver failure (Moreau et al., 2020). Thus, reduced PTV may also indicate an impaired autonomic thermoregulatory response to an imbalance between heat production and heat loss in patients with poor prognosis. In the present study, we quantified the memory length of patients’ temperature time series in order to indirectly assess the controllability of these temperature signals. A system is controllable if, with a choice of inputs, it can be driven from its initial state to a desired state within a finite time (Liu, Slotine, & Barabási, 2011). Intuitively, it is also harder to control a system with prolonged memory (Ghafari, Ghafari, Mani, & Raoufy, 2017; Mazloom et al., 2014). Controllability is reduced in a system with prolonged memory (Mazloom et al., 2014), thus the increased memory length exhibited in the nonsurviving patient group may be indicative of the decreased controllability of the autonomic thermoregulatory system in those with poorer prognosis. Within this context, an increased memory length can be a disadvantage for an adaptive system, a phenomenon which is reflected in the prognostic value of memory length in our patient population.

Recent studies have shown that the addition of physio‐markers to MELD, that is, EEG or HRV exhibit greater prognostic capacity than MELD alone. This corroborates with our work, as our data introduce PTV as a novel physio‐marker for predicting mortality in patients with cirrhosis. Survival prediction is immensely important in patients with cirrhosis, as their prognosis is the basis of liver transplant allocation. Currently, the MELD score has been used in recent years as a prognostic indicator in patients with cirrhosis. Physio‐markers such as PTV are shown to be independent of MELD in predicting survival. Therefore, conventional scoring systems such as MELD have indeed been now recognized as being limited in reflecting the multisystem nature of liver cirrhosis. Nonetheless, the addition of all plausibly efficacious physio‐markers such as PTV indices as well as HRV indices used together with MELD may create an ideal index. Hence, this novel index may indeed be more reflective of the physiological network that is altered during chronic liver disease due to the multisystem manifestations exhibited in cirrhosis. However, further studies are required to delineate the potential efficacy of this novel index, especially with a larger sample size (i.e., for multivariate Cox regression analysis or Competing risk analysis) or the development of novel techniques using a network physiology approach may also be feasible (Bartsch, Liu, Bashan, & Ivanov, 2015; Bashan, Bartsch, Kantelhardt, Havlin, & Ivanov, 2012).

Reduced HRV, specifically long‐term HRV indices (i.e., SD2) delineated from 10‐min ECG recordings taken from patients with cirrhosis, has been found to predict survival (Bhogal et al., 2019; Satti et al., 2019). Our data corroborate previous reports such as this, as this study reveals that HRV calculated from 24‐hr ECG recordings taken from cirrhotic patients also predicts mortality. Specifically, the long‐term fractal exponent (α2) also indicative of long‐term HRV calculated from these 24‐hr ECG recordings exhibits a prognostic value independently of MELD or Child‐Pugh. Furthermore, as the fluctuations of core body temperature affect one's cardiac rhythm, some investigators have postulated that long‐term HRV is due to inherent fluctuations of core body temperature (Fleisher et al., 1996). Firstly, this may plausibly be due to the rationalization that the heart fluctuates faster than one's core body temperature. Secondly, in response to an increase in core body temperature, cardiac ion channels are more active, and the diffusion rate of calcium is greater, resulting in increased action potential generation and hence heart rate. Therefore, short‐term fluctuations in core body temperature may indeed be inducing a greater degree of heart rate variability. In this study as both HRV and PTV predict mortality in our patients, we wonder whether or not reduced long‐term HRV in cirrhosis reflects decreased short‐term PTV. Although this hypothesis linking reduced short‐term PTV with long‐term HRV seems reasonable at first glance; bivariate Cox regression analysis revealed that these PTV and HRV indices are in fact independently associated with poor prognosis. Autonomic nervous system has diverse and complex functions and there is no single index to measure its activity. Our report showed that HRV and PTV predict mortality independently of each other. This probably indicates that each index measures an important aspect of autonomic control from a unique angle. It should be mentioned that we did not directly measure the variability exhibited in patients’ core body temperature, but, due to clinical feasibility, only the variability possessed in patients’ proximal skin temperature was measured and subsequently quantified. Thus, future studies may indeed explore this area further by delving deeper into the relationship between core body PTV and HRV in patients with chronic liver disease.

The limitations of this study are that the sample size and the power of the study are relatively small. In addition, this study only applies to hospitalized patients with cirrhosis who exhibit manifestations of decompensation. Thus, in the future, a bigger sample size and inclusion of a diverse group of patients with cirrhosis will further validate the true prognostic capacity of PTV analysis. Even though patients with documented infection (e.g., treated with antibiotics) were excluded, inflammation due to bacterial translocation or subclinical infections may play a role in altered PTV in nonsurvivors. This can be studied in future investigations. Furthermore, the effect of patients’ cutaneous circulation was not studied, despite how one's peripheral skin temperature measurement reflects a culmination of factors, such as the local blood‐flow rate, the extent of subcutaneous tissue beneath the iButton, as well as sympathetic input affecting the vascular tone. Although this questions the validity of utilizing skin temperature measurements, this is nonetheless the most clinically feasible approach. This is because the ingestion of telemetric probes to estimate patients’ core body temperature is challenging for cirrhotic patients, as they may require immediate medical intervention or imaging during their hospitalization. We measured temperature variability only in a 24‐hr period and do not know how PTV may change with time, severity of disease and development of complications. PTV appears to be a dynamic phenomenon and future longitudinal studies with multiple/long‐term temperature measurements can give more information on the true value of PTV monitoring in clinical practice.

To conclude, this study has revealed that proximal temperature fluctuation analysis is an independent physiological marker that can predict survival in hospitalized patients with cirrhosis.

## CONFLICT OF INTEREST

The authors declare that the research was conducted in the absence of any commercial or financial relationships that could be construed as a potential conflict of interest.

## Appendix 1

Extended Poincaré plots of patients’ proximal skin temperature time series. Poincaré plot used to study the correlation between Tn and Tn + k intervals is shown. (a) k = 1 (conventional Poincaré plot), (b) k = 3, (c) k = 10 (extended Poincaré plot). (d) Schematic diagram representing how the data points per extended Poincaré plot is derived.

## Appendix 2

ROC curves for MELD (a), extended SD1 (b) and memory length (c) in temperature variability analysis

## Appendix 3

Mean 24‐hr HRV characteristics of the study population.

**Table:**

	Survivors	Non‐survivors	*p* value
Mean heart rate (bpm)	76.6 ± 3.5	72.9 ± 3.0	.459
SDNN (ms)	86.79 ± 5.23	67.35 ± 6.46	.034
cSDNN	316.8 ± 22.6	236.0 ± 23.8	.020
SD1 (ms)	16.82 ± 2.79	26.25 ± 4.99	.112
SD2 (ms)	121.01 ± 8.37	90.25 ± 8.62	.016
VLF (ms^2^)	6,219 ± 852	3,315 ± 591	.011
LF (ms^2^)	269 ± 74	492 ± 209	.291
HF (ms^2^)	170 ± 49	420 ± 161	.154
Sample factory	0.640 ± 0.095	0.892 ± 0.117	.102
Short term scaling exponent (α_1_)	1.107 ± 0.062	0.940 ± 0.075	.093
Long term scaling exponent (α_1_)	1.142 ± 0.036	0.989 ± 0.032	.004

The data are expressed in mean ± SEM. Level of significance set at *p *< .05.

## Appendix 4

Mean proximal temperature during day (awake) and night (sleep) in survivors and nonsurvivors with cirrhosis. Data are expressed as mean ± standard deviation. A two‐way ANOVA did not show any significant effect for group or Day/Night on mean proximal temperature (F_day/night_ = 0.285, *p* = .596, F_group_ = 3.091, *p* = .085).
